# *EGFR*和*ROS-1*基因共突变非小细胞肺癌1例报道

**DOI:** 10.3779/j.issn.1009-3419.2025.102.26

**Published:** 2025-06-30

**Authors:** Juan ZHAO, Jiaofeng YU, Ye FU, Yan ZHAO, Mingli ZHAO

**Affiliations:** ^1^650500 昆明，昆明医科大学（赵娟，余娇凤，符叶，赵艳，赵明利）; ^1^Kunming Medical University, Kunming 650500, China; ^2^650118 昆明，昆明医科大学第三附属医院内三科（赵娟，余娇凤，符叶，赵艳）; ^2^Three Departments in the Third Affiliated Hospital of Kunming Medical University, Kunming 650118, China

**Keywords:** 肺肿瘤, *EGFR*, *ROS-1*, 共突变, 病例报道, Lung neoplasms, *EGFR*, *ROS-1*, Co-mutation, Case report

## Abstract

肺癌是当前世界上最常见的恶性肿瘤之一，其治疗已进入靶向治疗时代。表皮生长因子受体（epidermal growth factor receptor, *EGFR*）突变是非小细胞肺癌（non-small cell lung cancer, NSCLC）的常见基因突变类型，而*c-ros*原癌基因1受体酪氨酸激酶（*c-ros* oncogene 1 receptor tyrosine kinase, *ROS-1*）融合突变是NSCLC的罕见突变位点，目前关于合并*EGFR*和*ROS-1*基因突变共存的病例报道少见。本研究报道了1例合并*EGFR*和*ROS-1*基因突变共存的NSCLC病例，旨在为临床工作提供相关的治疗策略。

## Abstract (en)

Lung cancer represents one of the most prevalent malignant tumors globally, and its treatment has entered the era of targeted therapy. The epidermal growth factor receptor (*EGFR*) mutation is a common type of genetic mutation in non-small cell lung cancer (NSCLC), while *c-ros* oncogene 1 receptor tyrosine kinase (*ROS-1*) fusion mutation is a rare mutation site. Currently, there are few case reports on the coexistence of *EGFR* and *ROS-1* gene mutations. This study reports a case of NSCLC with coexisting *EGFR* and *ROS-1* gene mutations, aiming to provide relevant treatment strategies for clinical practice.

据国际癌症研究机构（International Agency for Research on Cancer, IARC）2024年公布的相关数据^[1]^，2022年全球肺癌新增病例数接近250万例，占癌症新增病例总数的12.4%，同时，有180万人死于肺癌，占2022年全球癌症死亡总数的18.7%。随着基因检测技术的进步和靶向治疗药物的研究和应用，肺癌患者的治疗进入靶向治疗时代，靶向药物的应用使得肺癌患者的生存率进一步提高。表皮生长因子受体（epidermal growth factor receptor, *EGFR*）突变是非小细胞肺癌（non-small cell lung cancer, NSCLC）的常见基因突变位点，*c-ros*肉瘤致癌因子-受体酪氨酸激酶（*c-ros* oncogene 1, receptor tyrosine kinase, *ROS-1*）突变以基因融合（重排）为主，占所有ROS1变异的绝大多数。在NSCLC中，*ROS-1*融合的发生率为1%-2%^[2]^。研究^[3,4]^发现，*ROS-1*突变与其他驱动基因互斥，极少与间变性淋巴瘤激酶（anaplastic lymphoma kinase, *ALK*）、Kirsten大鼠肉瘤病毒癌基因同源物（Kirsten rat sarcoma viral oncogene homolog, *KRAS*）或*EGFR*突变共存。*EGFR*和*ROS-1*突变共存的发生率低于0.6%^[5]^。目前关于合并*EGFR*和*ROS-1*基因突变共存的病例报道少见，本文报道了1例*EGFR*和*ROS-1*基因突变共存NSCLC病例，其使用埃克替尼联合克唑替尼治疗，无进展生存期（progression-free survival, PFS）达35个月，为临床工作提供了有效的治疗策略。现报道如下。

## 1 病例资料

患者男，56岁，因“咳嗽、咳痰2月”于2021年2月21日就诊于云南省某三甲医院，行计算机断层扫描（computed tomography, CT）检查提示右肺上叶多发实变，完善CT引导下右肺上叶实变区穿刺，组织病理类型为肺腺癌（图1）。后转诊至昆明医科大学第三附属医院。2021年4月28日完善CT提示：右肺多发实变、双肺多发粟粒结节，性质恶性可能；纵隔及左锁骨上多发肿大淋巴结，转移可能；肝多发低密度结节；右肾上腺区域结节；双侧基底节区低密度结节，性质倾向良性；余影像检查未见异常（图2）。予行肺癌相关敏感基因检测。诊断：右肺上叶腺癌并纵隔、左锁骨上多发淋巴结转移，双肺转移，cT4N2M1 IV期。等待基因检测结果期间，于2021年5月1日行培美曲塞+卡铂化疗1个周期，出现II级消化道反应，予止吐、止泻、保护消化道等对症处理后缓解。2021年5月6日行基因检测，检测方法为突变扩增阻滞系统（amplification refractory mutation system, ARMS），结果示：*EGFR*外显子21 L858R点突变、*ROS-1*融合突变（融合位点：外显子32；融合伴侣位点：CD74外显子6）。*EGFR*基因突变丰度较低，于2021年5月25日开始予患者埃克替尼125 mg *tid*联合克唑替尼250 mg *bid*靶向治疗。

**图1:**
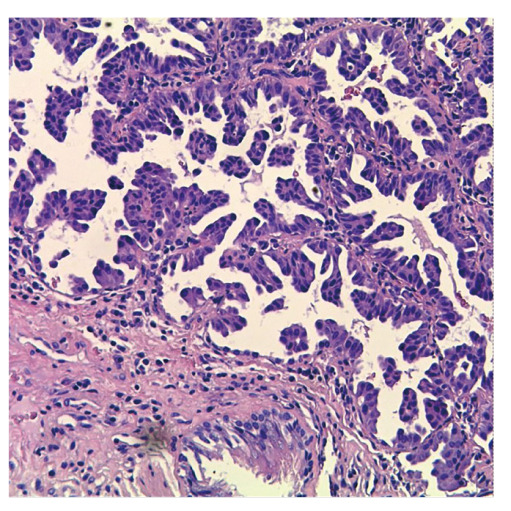
病理切片显示为肺腺癌（HE染色，×100）

**图2:**
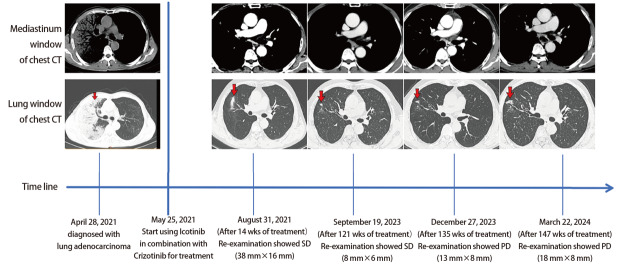
患者治疗经过及治疗期间重要节点影像学表现

治疗后定期复查CT（图2），2021年8月31日（治疗14周）评估疗效：病情稳定（stable disease, SD），后定期复查CT，至2023年9月19日（治疗121周）疗效评估均为SD，期间出现轻度消化道反应。直至2023年12月27日（治疗135周），患者CT提示病情有缓慢进展趋势，根据实体瘤疗效评估标准1.1（Response Evaluation Criteria in Solid Tumors 1.1, RECIST 1.1），评价疗效为病情进展（progressive disease, PD），期间出现轻度消化道反应。2024年3月22日（治疗147周），患者CT提示PD，PFS达35个月。

患者病情缓慢进展，建议行二次活检及基因检测评估是否存在如*EGFR* T790M、*ROS-1*继发突变等耐药机制，因双肺多发转移灶取材阳性率不高且合并有一定风险，患者拒绝二次取材；且因基因检测为自费且全基因的二代测序（next-generation sequencing, NGS）不能报销，患者仅同意行血液学的T790M检测，遂采血液行基因检测[检测方法：滴液式数字聚合酶链式反应（polymerase chain reaction, PCR）法]，结果提示：未检测到*EGFR*外显子20 T790M突变。经多学科会诊后，治疗以全身药物治疗为主，双肺多发转移灶无明显临床症状，局部治疗（手术、放疗、射频等）不获益。结合患者病情缓慢进展，肿瘤负荷低，症状不明显，于2024年4月12日至9月25日予卡铂（400 mg d1 q3w）化疗7个周期，期间出现中度肝功能损伤及III度骨髓抑制。追问病史，患者于治疗期间自行口服中药治疗（具体成分不详），且换用贝伐珠单抗期间肝功能改善不明显，故考虑患者肝功能损伤与中药有关。治疗期间评估疗效，均为SD；治疗7个周期后因骨髓抑制明显，而于2024年10月22日行贝伐珠单抗方案（贝伐珠单抗500 mg d1 q3w）单药维持治疗1次，出现中度肝功能损伤、轻度消化道反应，后复查疗效评估SD。2025年3月3日患者CT提示病情缓慢进展，于2025年3月12日行“贝伐珠单抗+培美曲塞+卡铂”联合化疗（贝伐珠单抗500 mg d1，培美曲塞800 mg d1，卡铂400 mg d2 q3w），期间出现轻度消化道反应，予止吐、止泻、保护消化道等对症处理后缓解。

## 2 讨论

对于亚洲的NSCLC病例而言，*EGFR*基因突变是比较常见的突变类型，其发病率为38.4%-49.1%^[6]^。并且，*EGFR*-酪氨酸激酶抑制剂（*EGFR*-tyrosine kinase inhibitors, EGFR-TKIs）在治疗*EGFR*突变的患者时，有着一定的疗效。目前国内获批上市的EGFR-TKIs药物分为三代，第四代药物正开展相关临床研究。埃克替尼属于第一代靶向药物，也是我国第一个拥有自主产权的EGFR-TKIs药物，与同类型药物相比具有价格优势、安全性好的特点。*ROS-1*突变类型包括融合、扩增、重排、点突变等，其中，融合是*ROS-1*基因主要变异类型。研究^[2]^表明，*ROS-1*融合突变在NSCLC人群中的发生率为1%-2%。在NSCLC中*ROS-1*基因主要与CD74、SLC34A发生融合。研究^[2]^表明，*ROS-1*重排的患者有以下几个临床特点：年轻、不抽烟、肺腺癌、女性、晚期淋巴结分期。目前针对*ROS-1*突变的NSCLC患者，经美国食品药品监督管理局（Food and Drug Administration, FDA）批准，克唑替尼、恩曲替尼和瑞普替尼等药物已成为首选的一线治疗方案。

既往研究^[3,4]^表明，NSCLC患者的主要驱动基因多存在互斥性，包括*EGFR*、*ALK*、*KRAS*、人表皮生长因子受体2（human epidermal growth factor receptor 2, HER2）和鼠类肉瘤病毒癌基因同源物（V-Raf murine sarcoma viral oncogene homolog B, *BRAF*）等。但近年来，也有研究发现*EGFR*和*ROS-1*突变并非互斥关系，在小部分研究中也有报道*EGFR*与*ROS-1*突变共存的情况^[7-9]^。相较于*EGFR*分别与*ALK*、*KRAS*及*MET*基因发生突变共存（发生率分别为4%-14%、6%-36%及23%-29%），*EGFR*和*ROS-1*突变共存的发生率低于0.6%^[5]^。

目前关于NSCLC基因突变共存的发生机制，涉及信号通路的交互影响、肿瘤异质性、基因组不稳定性等多个方面。研究^[10]^表明，不同的遗传变异可能发生在不同的肿瘤细胞中，基因和表型异质性可能源于肿瘤细胞的遗传不稳定性，进而容易导致多种基因突变和染色体的重排现象。同时，也有不同的驱动基因变异发生在同一肿瘤细胞克隆内的可能，且可能共同推动肿瘤的进展^[11]^。根据现有研究^[12]^，目前存在以下几种可能：（1）肿瘤组织内的不同细胞可能会出现多样的基因突变；（2）肿瘤组织中的同一细胞也可能出现多种不同的基因突变；（3）肿瘤组织在原发灶和转移灶的位置上，同样可能出现不同的基因突变情况。

目前对于*EGFR*合并*ROS-1*突变共存病例少见，在患者靶向治疗药物的选择策略方面，目前仍缺乏统一标准。在现有资料中，*EGFR*合并*ROS-1*突变共存的治疗策略有以下几种：（1）单纯使用EGFR-TKIs治疗。Alfayea等^[8]^报道了使用奥希替尼治疗1例*EGFR*突变（位点：L858点突变；丰度：29%）和*ROS-1*融合（融合位点：*SLC32A2*；丰度：22%）的肺腺癌患者，在此方案治疗下，该患者SD超过3年。除此之外，1例*EGFR*（位点：G719V；丰度：8.4%）、*ROS-1*（融合位点：*SLC34A2*；突变丰度不详）、*BRAF*多靶点突变的肺腺癌患者在使用奥希替尼治疗后，SD大于2个月^[8]^。在Lambros等^[13]^的报道中，10例使用EGFR-TKIs治疗的*EGFR*及*ROS-1*突变共存患者中，疗效评价为部分缓解（partial response, PR）6例，SD 2例，PD 2例，但文中未记录患者的具体突变位点及丰度情况。（2）单纯使用克唑替尼治疗。Zhang等^[14]^描述了1例携带*EGFR*突变（位点：20ins；丰度：0.21%）、ALK融合（融合位点：*EML4*；丰度：3.81%）和*ROS-1*融合（融合位点：*MAGI3*；丰度：0.2%）突变共存的晚期肺腺癌患者，首先使用克唑替尼治疗，取得了28个月的PFS。（3）EGFR-TKIs联合克唑替尼同时治疗。Wu等^[15]^报道了1例*EGFR*突变（位点：19del；丰度：14.38%）合并*ROS-1*融合突变（融合位点：SLC34A2；丰度：5.93%）的肺腺癌患者，予以口服阿美替尼和克唑替尼联合治疗，1个月后疗效评估为PR。（4）先后分别予EGFR-TKIs及克唑替尼治疗。Guo等^[16]^认为针对*EGFR*及*ROS-1*基因突变共存患者，克唑替尼作为EGFR-TKIs进展后的二线治疗可能更有用。而在Peng等^[7]^的报道中，1例*EGFR*突变（位点：L858R；丰度：1.3%）和ROS1融合（融合位点：EZR；突变丰度未提及）的肺腺癌患者，在使用吉非替尼治疗1个月后，完善相关检查评估为PD，予二线克唑替尼治疗，患者耐受情况良好，PFS长达53个月，这也是目前报道的*EGFR*及*ROS-1*突变共存患者的最长PFS。在以上治疗方式的应用中，不同患者所对应的疾病控制时间跨度较大，且需要综合考量突变位点如何覆盖及联合或序贯治疗存在的毒副反应问题，故针对*EGFR*合并*ROS-1*突变共存的病例，目前暂无标准治疗方案。目前关于*EGFR*及*ROS-1*突变共存患者治疗策略的选择依然是一个极大的挑战，尽管目前尚无明确的共识指导这类患者的治疗，但有研究^[8]^强调了在选择一线治疗方案时需考虑突变丰度，即较高的突变丰度对于治疗选择有提示意义。由于合并敏感基因突变共存的罕见和复杂，针对*EGFR*和*ROS-1*突变共存患者，个体化治疗显得十分重要。未来的研究将更加专注于针对*EGFR*和*ROS-1*突变共存患者的治疗模式选择，旨在验证不同治疗方案的疗效与安全性，并确定最优的治疗模式和药物组合。此外，相关研究还需深入挖掘这类患者身上其他的潜在治疗靶点，着力开发新型靶向药物，以便为患者创造更多的治疗机会。

根据现有研究，针对*EGFR*和*ROS-1*突变共存的患者，双靶治疗可延长患者生存期，部分患者可获得长期SD^[7,16]^，但由于每位患者的基因突变丰度、肿瘤生物学行为等存在差异，双靶联合治疗的疗效也因人而异。此外，双靶治疗也可能存在一些潜在的不良反应风险，除外常见的消化道反应和皮肤反应，EGFR-TKIs和克唑替尼均会导致患者出现高血压、心脏功能障碍等心血管不良反应，也会出现肝酶升高等肝功能异常的情况^[17]^。目前，关于EGFR-TKIs与克唑替尼联合或序贯治疗的毒副反应研究较为有限，这种联合或序贯治疗可能会增加消化道反应、心血管毒性、肝毒性等不良反应的发生风险，从而带来更为复杂的毒副反应问题。因此，在临床实践中，医护人员需要密切监测患者的不良反应情况，并根据患者的个体状况，适时调整治疗方案，以确保治疗的安全性和有效性。

综上所述，本文报道了1例合并*EGFR*和*ROS-1*基因突变共存的NSCLC病例，患者使用埃克替尼联合克唑替尼双靶向治疗后，PFS为35个月，毒副反应可耐受，未出现新发的不良反应。现在对于携带*EGFR*和*ROS-1*基因突变患者的治疗，因缺乏大量临床数据而存在诸多未解问题，亟待进一步思考和研究，其中包括靶向药物的运用，以及靶向药物与化疗或免疫治疗的联合使用能否达成更优疗效等。该病例报告为合并基因突变共存的NSCLC提供了治疗经验，未来研究可聚焦于基因突变共存的分子机制及联合靶向治疗的优化策略。
